# A Comparison of Different Informative Vibrotactile Forward Collision Warnings: Does the Warning Need to Be Linked to the Collision Event?

**DOI:** 10.1371/journal.pone.0087070

**Published:** 2014-01-27

**Authors:** Rob Gray, Cristy Ho, Charles Spence

**Affiliations:** 1 School of Sport, Exercise and Rehabilitation Sciences, University of Birmingham, Birmingham, United Kingdom; 2 Department of Experimental Psychology, University of Oxford, Oxford, United Kingdom; University of California, Merced, United States of America

## Abstract

Recent research demonstrates that auditory and vibrotactile forward collision warnings presenting a motion signal (e.g., looming or apparent motion across the body surface) can facilitate speeded braking reaction times (BRTs). The purpose of the present study was to expand on this work by directly comparing warning signals in which the motion conveyed was constant across all collision events with signals in which the speed of motion was dependent on the closing velocity (CV). Two experiments were conducted using a simulated car-following task and BRTs were measured. In Experiment 1, increasing intensity (looming) vibrotactile signals were presented from a single tactor attached to the driver's waist. When the increase in intensity was CV-linked, BRTs were significantly faster as compared to a no-warning condition, however, they were not significantly different from constant intensity and CV-independent looming warnings. In Experiment 2, a vertical array of three tactors was used to create motion either towards (upwards) or away (downwards) from the driver's head. When the warning signal presented upwards motion that was CV-linked, BRTs were significantly faster than all other warning types. Downwards warnings led to a significantly higher number of brake activations in false alarm situations as compared to upwards moving warnings. The effectiveness of dynamic tactile collision warnings would therefore appear to depend on both the link between the warning and collision event and on the directionality of the warning signal.

## Introduction

In recent years, there has been a great deal of interest in the development of driver assistance systems, in particular collision warnings [1], [2]. These systems present a low-cost solution to reducing the substantial loss of life, serious injuries, and financial costs associated with driving worldwide. Research in this area has demonstrated that auditory, tactile, and multisensory warning signals can effectively be used to *alert* a driver to a potential danger and to *orient* their attention to the relevant location/direction (as reviewed in [3]). However, these warnings typically do very little to *inform* the driver about the nature of the collision event (e.g., the urgency/severity of the situation). For a collision warning to be effective, it must rapidly elicit the appropriate behavioural response from the driver. It is for this reason that some researchers have proposed using informative symbolic warnings (or icons) that bear some relationship between the signal and the collision event.

Indeed, warning signals such as the sound of a car horn, verbal signals such as the word “danger”, or screeching tires have been shown to lead to faster driver brake reaction times (BRTs) to a potential collision than abstract warning signals that are comprised of pure tones [4]. However, these symbolic warnings can sometimes come at a cost: Drivers may make more inappropriate braking responses in false alarm situations. Furthermore, in more realistic driving conditions, it is likely that these types of warnings may be confused with other sounds that may be present in the environment [5] or else be interfered with by the concurrent linguistic processing involved in listening to the radio, talking to a passenger or on a cell phone while driving [6].

An alternative approach to using symbolic warnings has been to develop more complex abstract signals that both inform and alert a driver. For example, it was recently demonstrated that *auditory looming warnings* (specifically, sounds whose intensity increased as the distance between the driver's vehicle and the lead vehicle decreased) resulted in faster BRTs of a magnitude that was equivalent to an (abrupt) car horn warning but with very low rates of false alarm braking [7]. These warning signals are informative to the driver because they take advantage of the natural mapping: As a sound-emitting object approaches an observer, there is an associated increase in sound intensity (looming) and the rate of change of intensity will be related to the time to collision (TTC), that is, it will signal urgency [8]. Auditory looming and its visual analogue (i.e., a rate of increase in angular size of an approaching object, commonly known as *tau*) provide very powerful signals to the human perceptual system. Research has shown that looming signals induce defensive reactions in both adults and infants [9], [10], and that they also capture attention [11].

A further advantage of an auditory looming warning is that its signal properties can easily be altered to shorten the TTC signalled by the warning (i.e., to increase the signal's perceived urgency) [7]. When the rate of change of acoustic intensity was increased (consistent with a TTC earlier than the actual TTC), BRTs were significantly faster than for a veridical warning. Conversely, warnings that signalled a later TTC than the actual TTC led to significantly slower BRTs. This occurred despite the fact that visual information about the TTC with the lead vehicle (i.e., its rate of expansion) was identical in all conditions. These findings therefore suggest that the acoustic signal was attributed to the approaching vehicle and directly altered the driver's perceived TTC.

Can analogous informative collision warnings be developed using vibrotactile signals? This is an important question to investigate given that it has been proposed that tactile warnings may be more effective than equivalent warning signals presented in other sensory modalities [3]. This is because the sense of touch is less involved in the task of driving [3] and any other secondary tasks that a driver may engage in, such as talking on a phone or listening to music, as compared to vision or audition (e.g., [12]–[13]). For example, it has been shown that abstract auditory collision warnings are rendered ineffective by the addition of a phone conversation whereas abstract tactile warnings still produce significantly reduced BRT [6].

However, there is a potential difficulty associated with conveying information about an impending potential collision using vibrotactile signals, namely the sense of touch typically conveys information about objects in peripersonal space (i.e., the area immediately surrounding our body) whereas driver assistance systems need to warn a driver about an event in extrapersonal space (i.e., far from the body). Can proximal stimulation via a vibrotactile signal on the skin surface be used to convey information about a distal event such as a potential collision with a vehicle that is several meters from the driver's body? Recent research suggests that indeed this may be possible as proximal vibrotactile stimulation can result in distal attribution to far objects in the perception of object form [14], distance [15], and can even be used to guide locomotion while blindfolded [16].

In our recent research, we have investigated two types of vibrotactile collision warning signals: A looming intensity signal analogous to the auditory looming warning described above (Experiment 3 [17]) and signals that were designed to generate a sensation of tactile apparent motion on the abdomen [18]. For the vibrotactile looming warning, in contrast to comparable results for auditory looming signals [7], we did not find any evidence of an informative effect as BRTs were not significantly different for the looming vibrotactile signal and other non-looming vibrotactile warnings including constant-intensity, pulsed, and ramped intensity signals. In the apparent motion study, we found faster BRTs for the sequential stimulation of three tactors aligned vertically on the abdomen as compared to equal duration signals using one or two tactors. However, this was the case for all orders of stimulation of the three tactors. In other words, the effectiveness of the warning signal did not appear to rely on perceiving the apparent motion of the constituent signals, thus suggesting that the effect was instead attributable to improved attentional re-orienting. Therefore, our initial findings are somewhat unclear as to whether it is possible to convey information about a collision event using vibrotactile warnings in the same manner as has been shown previously for auditory warnings.

Two possible explanations for the lack of performance facilitation following these vibrotactile warnings relates to: (i) the direction of motion simulated and (ii) the coupling between the warning and the collision event. In our previous study described above [18], 1-D apparent motion (i.e., motion across the surface of the abdomen either towards or away from the head) was used to warn the driver about an event involving primarily 3-D motion (i.e., approaching/approach of another vehicle). Given that it has been shown that frontal-plane motion (i.e., 1-D or 2-D) is processed relatively independently from 3-D motion in the human brain (e.g., [19]), it is perhaps not surprisingly that our 1-D warnings did not effectively inform drivers about a 3-D event.

A second potentially important limitation of our previous studies was that, unlike auditory looming warnings [7], the vibrotactile warnings were not linked to parameters of the collision event. For example, the looming vibrotactile signal did not increase at a faster rate and the apparent motion signals did not have a higher speed when the closing velocity was higher. Instead, the parameters remained constant across all possible collision events. Previous research has shown that distal attribution is stronger for proximal vibrotactile stimulation when the stimulation is contingent on the actions that are being performed by the wearer as compared to non-changing (static) patterns [16]. Therefore, it is likely that vibrotactile collision warnings that are linked to the collision event will be more effective.

The goal of the present study was to investigate the plausibility of this second explanation (i.e., that the previous vibrotactile warnings we have used were relatively ineffective because they were not linked to the collision event). In a separate set of studies, we have been evaluating the first explanation by comparing 1-D vibrotactile warnings with signals that attempt to convey 3-D motion [20].

In Experiment 1 of the present study, vibrotactile signals were presented from a single tactor that was fastened to the driver's waist. A CV-linked looming warning was compared with a CV-independent looming warning, a constant intensity warning and a no warning condition. For the CV-linked warning, the rate of increase of vibrotactile intensity was proportional to the CV between vehicles. We predicted that the presentation of CV-linked warning signals would lead to significantly faster BRTs than all of the other types of warning signal. In Experiment 2, a vertical array of three tactors was used to create motion upwards (towards) or downwards (away) from the driver's head (similar to [18]). CV-linked upwards and downwards warnings were compared with CV-independent warnings and a no warning condition. For the CV-linked warnings, the inter-tactor interval was inversely proportional to the CV; simulating a faster rate of apparent motion. We predicted that the CV-linked upwards motion warning would lead to significantly faster BRTs as compared to the other conditions.

## Experiment 1

### Participants

Sixteen participants (9 male, 7 female; mean age of 26.2±3.1 years) were recruited from the University of Birmingham campus. Participants received payment of £10 for their participation. All of the participants had a full valid UK driving license with between 2 and 9 (mean = 4.2±2.7) years driving experience. Participants were asked to wear any prescribed lenses (i.e., glasses or contacts) during testing.

### Ethics Statement

The work reported here was approved by the Science, Technology, Mathematics, and Engineering (STEM) Ethical Review Committee at The University of Birmingham and adhered to the Declaration of Helsinki. All participants signed a consent form.

### Methods

#### Driving Simulator

The XPI Simulation Limited™ XPDS-XP300 driving simulator (version 2.2) was used. The simulator was comprised of a Logitech G25 Racing Wheel/Pedals and three Microsoft Plug and Play monitors with 43.2 cm displays (2840×1025 pixels resolution). The system ran using the NVIDIA GeForce GTS 450 graphics card with a 1024 MB memory. Participants positioned themselves so that they could comfortably use the steering wheel and pedals and such that their eyes were 80 cm away from the computer monitors.

#### Collision Warning Signals

Three different vibrotactile warning signals were used: CV-independent looming, CV-linked looming, and constant intensity. All of the vibrotactile stimuli were presented via a single tactor (VBW32, Audiological Engineering Corp., Somerville, MA, USA) fastened to the participant's waist using a Velcro belt. These warning signals were compared with a condition in which no warnings were given. All of the warning signals were delivered at a frequency of 250 Hz, and had a duration of 1 s. All of the warnings were activated on the basis of the same parameters (i.e., when the TTC with the lead vehicle fell below a certain critical threshold, as described below). The warning signals only differed in the nature of the signal after activation. The constant intensity warnings had an intensity that was half of the full intensity of the tactor. The intensity remained the same after the warning had been activated until it was terminated.

The CV-independent looming vibrotactile warning was identical to that used in our previous study (Experiment 3 [17]). The warning signal began at two-ninths of the full intensity of the tactor and ended at seven-ninths of the full intensity. The intensity (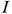) was updated according to the formula 
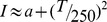
where 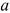 was the initial intensity and 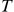 was the time (in ms) from the onset of the warning.

The CV-linked looming virbotacile warnings were modelled after the auditory looming warnings we developed previously [7]. The warning signal was based on the following two equations:
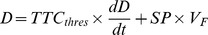
(1)

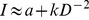
(2)


In equation 1, *D* was the distance from the lead vehicle at which the warning was activated, *dD/dt* was closure rate (which is determined by the speeds of both vehicles) and *V_f_* was the following vehicle's speed. *S_P_* (speed penalty) and TTC*_thres_* (time to collision threshold) were values that could be set within the system. In the present study, the recommended value of 0.4905 was used for the speed penalty [21]. The essential goal of this penalty parameter was to warn the driver earlier when they are travelling at a higher approach velocity (thus requiring a greater stopping distance). Two different TTC*_thres_* values were used: 3 s (the recommended value) and 7 s. The 7 s value was used to create “false alarm” situations in which the alarm was activated for an event that did not require the driver to respond with a braking manoeuvre. In equation 2, *a* and *k* were constants with values of 0 and 150000. These values were chosen to make the intensity of the warning approximately two-ninths of the full intensity of the tactor at a simulated distance of 100 m (the largest distance at which drivers received a warning in the present study) and to ensure that the intensity level was never greater than seven-ninths of the full intensity. Note that all of these values are analogous to the auditory signals used in [7].

### Design and Procedure

The driving test involved car following scenario similar to that used in our previous studies (e.g., [7], [12], [21]). On each trial, the lead vehicle began from a stationary position and accelerated to a speed of 60 mph. It then travelled at a speed ranging between 55 and 65 mph with speed changes once every 5 sec, on average. At a random time interval (between 60–180 s after the beginning of the trial) the lead car braked suddenly with a −6 m/s^2^ deceleration rate. The drivers were instructed to accelerate in order to catch up with the lead vehicle and then maintain a 2 s time headway (TH). If the drivers followed too far behind the lead car, the phrase “*Speed Up!*” was presented over a loudspeaker. There was no analogous “*Slow Down!*” warning, so drivers were free to maintain any TH below 2.0 s. The brake lights of the lead vehicle were deactivated to allow for comparison with our previous studies (e.g., [12], [22]). Drivers were further instructed that they must brake to avoid collision with the lead vehicle and must not go out of the lane (any trials for which this occurred were discarded and re-run). Each trial ended when the participant's car came to a complete stop and/or collided with the lead vehicle. On a small proportion of the trials, the lead vehicle did not brake suddenly and the trial ended after 180 sec. These trials were included to reduce the tendency of drivers to begin to anticipate the stopping event near the end of trials.

Each driver completed 4 blocks of 20 trials; one block for each warning condition. The 20 trials were comprised of 14 events in which the warning (if present) was reliable (i.e., warning activated at TTC = 3 s), 4 trials in which the warning was unreliable (i.e., warning activated at TTC = 7 s), and 2 events in which the lead car did not stop. In the two vibrotactile warning conditions, the drivers were given the following instructions (analogous to those used in [7]):

In this condition, you will feel a vibration on your seat belt indicating that you are about to collide with the vehicle in front. Please use this warning signal in any manner you wish to help avoid a collision. For example, when you detect the warning, you may choose to let off the accelerator, slam on the brake, or do nothing at all.

During each block the warning type remained the same (i.e., we did not interleave different warning conditions). The order of blocks was partially counterbalanced across participants. In particular, we ensured that each warning condition was presented first and last for an equal number of participants.

#### Data Analysis

The primary dependent variable was the BRT which was defined as the elapsed time between the TTC with the lead vehicle falling below the critical threshold of 3.0 s and the driver depressing the brake pedal. Note that data from the false alarm conditions were not included in the calculation of BRT. For each driver, the mean BRT was based on 14 repeats for each warning conditions. BRTs were first analysed using a one-way repeated measures ANOVA with warning condition as the factor. We next performed planned comparisons between the warning we predicted would result in the best driving performance (i.e., CV-Linked) and all other conditions.

A secondary dependent variable was the total number of false alarm, which was defined as an instance in which the brake force reached >50% of the maximum force within 1 s of the warning onset with a TTC threshold of 7 sec. These variables were analysed with separate one-way ANOVAs.

### Results

**Figure 1:**
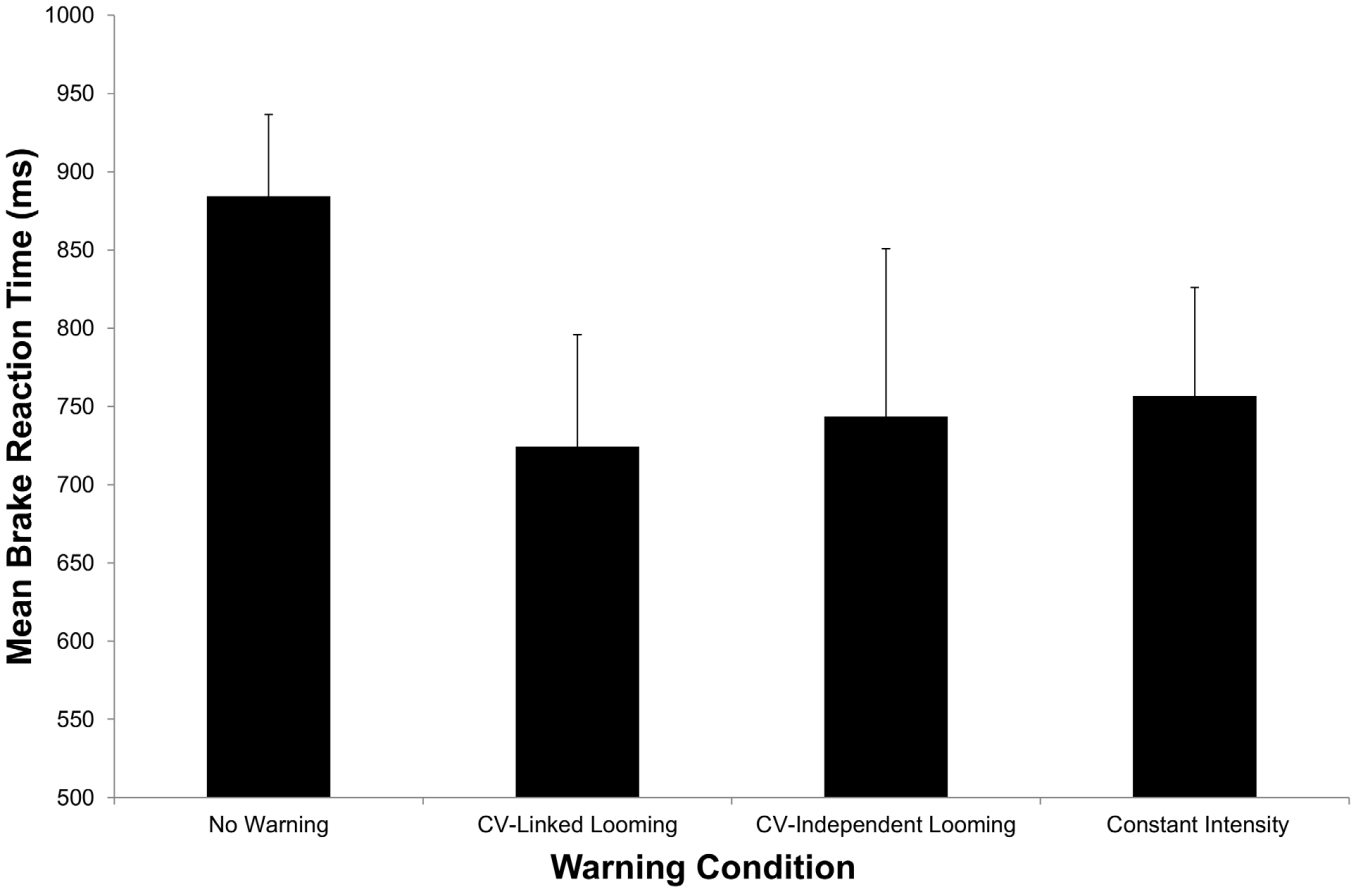
Mean braking reaction times for Experiment 1. Error bars are standard deviations.

Figure 1 shows the mean BRT for each of the four experimental conditions. The one-way ANOVA performed on these data revealed a significant effect of warning type: *F*(3, 45) = 11.9, *p*<0.001, 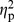 = .46. The planned comparisons revealed that BRTs were significantly faster in the CV-Linked condition than in the No Warning condition: *t*(15) = 6.4, *p*<0.001. None of the other planned comparisons was significant (all *p*s>0.1). Post-hoc comparisons, with Bonferroni correction for type I error (p = 0.025), revealed that BRTs for the CV-independent looming warning were significantly shorter than in the no warning condition [t(14) = 5.0, p<0.001] and that BRTs for the constant intensity warning were significantly shorter than in the no warning condition [t(14) = 4.9, p<0.001].

The mean total number of false alarm brake activations (out of a maximum possible of 2) for the warning signals were: CV-linked looming, 0.26 (*SD* = 0.41); CV-independent looming, 0.33 (*SD* = 0.35); constant intensity, 0.40 (*SD* = 0.42). Note that this variable cannot be calculated in the no warning condition. The one-way ANOVA performed on these data revealed a non-significant effect of warning type (*p* = 0.23).

### Discussion

We had predicted that a CV-linked vibrotactile looming warning would lead to significantly faster BRTs as compared to a CV-independent looming warning and a non-looming vibrotactile warning. This prediction was based on our previous findings ([7], [17] - Experiments 1 & 2) in which we found this pattern of results for comparable auditory warnings and experiments showing that distal attribution is greater for action-contingent vibrotactile stimulation [16]. The results of Experiment 1 of the present study did not support this prediction as there was no significant difference in BRTs among the different warning types. All warnings resulted in roughly the same decrease in BRT relative to the no warning condition.

So why do looming vibrotactile signals not result in the same BRT benefits as comparable auditory signals? One possible explanation here is that the participants in our studies did not attribute the proximal vibrotactile stimulation to the distal event of an approaching vehicle. This is consistent with the subjective impressions of participants as the majority indicated that the vibrotactile signal did not give the impression of an approaching object. In Experiment 2, we explored this possibility further by testing a different type of informative vibrotactile signal.

## Experiment 2

### Rationale

In Experiment 1, we investigated whether the effectiveness of a “looming” vibrotactile warning presented from a single tactor [17] could be improved by linking the warning signal and the collision event. In Experiment 2, the goal was to perform a comparable investigation for vibrotactile apparent motion warnings [18]. This experiment was designed to test two primary hypotheses. First, we predicted that brake RTs would be significantly shorter for CV-linked warnings as compared to CV-independent warnings (for both movement directions); a prediction again based on previous research showing distal attribution for action-contingent signals.

The second goal of Experiment 2 was to compare vibrotactile apparent motion warning signals that moved towards the driver's head versus warning signals that moved away from the driver's head. Previous research has provided several findings to suggest these two signals might produce different responses. First, it has been shown that we tend to judge the spatial location of ourselves and other objects in the environment relative to our head (precisely, midway behind the eyes, cf. [23]–[24]) as opposed to other body parts. Second, we are more sensitive (i.e., we exhibit the lowest directional discrimination thresholds) for visual motion that is head-on as compared to motion in other directions [25]. Finally, auditory neuroscience research has demonstrated that auditory events moving toward a person have greater salience than those that are moving away (e.g., [11], [26]). Based on these previous findings, we predicted that the up CV-linked warning signals would produce significantly faster BRTs as compared to the down CV-linked warning signals.

Note that in Experiment 2, apparent motion was generated across the driver's abdomen (i.e., 2D motion). We chose this type of apparent motion for two reasons: First, in terms of application, it was easier to generate a consistent direction of apparent motion on the driver's abdomen as compared to the arms or hands, for example, as the latter body parts frequently change location while driving. Second, we wanted to allow for direction comparison between the present results and our previous study using apparent motion on the abdomen [18]. In this previous study, we found no significant difference in BRTs for upwards versus downwards motion warnings. However, all warnings in this previous study were CV-independent, therefore, we wanted to rule out that factor as a possible explanation for the results. As mentioned above, in a separate set of studies we are currently investigating the potential of using 3D apparent motion signals for collision warnings.

### Participants

Fifteen participants (8 male, 7 female; mean age of 25.1±2.9 years) were recruited from the University of Birmingham campus. The participants received payment of £10 for their participation. All of the participants had a full valid UK driving license with between 1.5 and 7 (mean = 3.8±1.8) years of driving experience. Participants were asked to wear any prescribed lenses (i.e., glasses or contacts) during testing.

### Ethics Statement

The work reported here was approved by the Science, Technology, Mathematics and Engineering (STEM) Ethical Review Committee at The University of Birmingham and adhered to the Declaration of Helsinki. All participants signed a consent form and none had participated in Experiment 1.

### Methods

#### Apparatus

The driving simulator and testing procedure were identical to that used in Experiment 1.

#### Collision Warning Signals

Four different vibrotactile warning signals designed to generate apparent motion across the body were used: up CV-linked, up CV-independent, down CV-linked, and down CV-independent. These warning signals were compared with a condition in which no warnings were given. All of the vibrotactile stimuli were presented via three tactors (VBW32, Audiological Engineering Corp., Somerville, MA, USA) fastened to the participant's abdomen using a Velcro belt. The tactors were aligned vertically with the distance between adjacent tactors being 23 mm center-to-center such that the tactors were close but not touching one another. The tactors were driven by a 250 Hz sinusoidal signal at 1/4 of the full intensity of the tactor that was clearly perceivable by our participants. For the “up” signals, the tactor furthest from the participant's head was activated first, followed by the middle tactor then the tactor closest to their head. The “down” signals were presented in the opposite pattern.

For the two CV-independent signals, the tactors were operated sequentially, each for 215 ms for a total warning duration of 645 ms. The next tactor in the sequence was activated immediately the previous tactor was turned off (i.e., there was no inter-tactor interval, where this interval was defined as the time between offset and onset of adjacent stimuli). Note that the 645 ms value was chosen so that it matched the mean duration for CV-linked warnings (see below). These warning signals are similar to those used in our previous study [18] with the only difference being a longer duration.

For the two CV-linked signals, the activation of each tactor in the sequence was constant at 150 ms but the inter-tactor interval (ITI) was dependent on the CV. Specifically, ITI was given by:
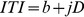
(3)


In this equation, 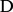 was determined by equation 1 while 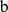 and 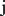 were constants with values of −550 and 0.007 (determined through pilot experiments). The net result of this manipulation was that the ITI was shorter at higher closing velocities, giving rise to a perception of faster apparent motion. For the car following speeds used in the present study, the ITI varied between roughly 50–150 ms while the total duration of the warning signal varied between roughly 550–750 ms.

#### Data Analysis

The same dependent variables and statistical analyses used in Experiment 1 were used. Planned comparisons were again made between the warning we predicted would result in the best driving performance (i.e., up CV-linked warning) and all other conditions.

### Results

**Figure 2:**
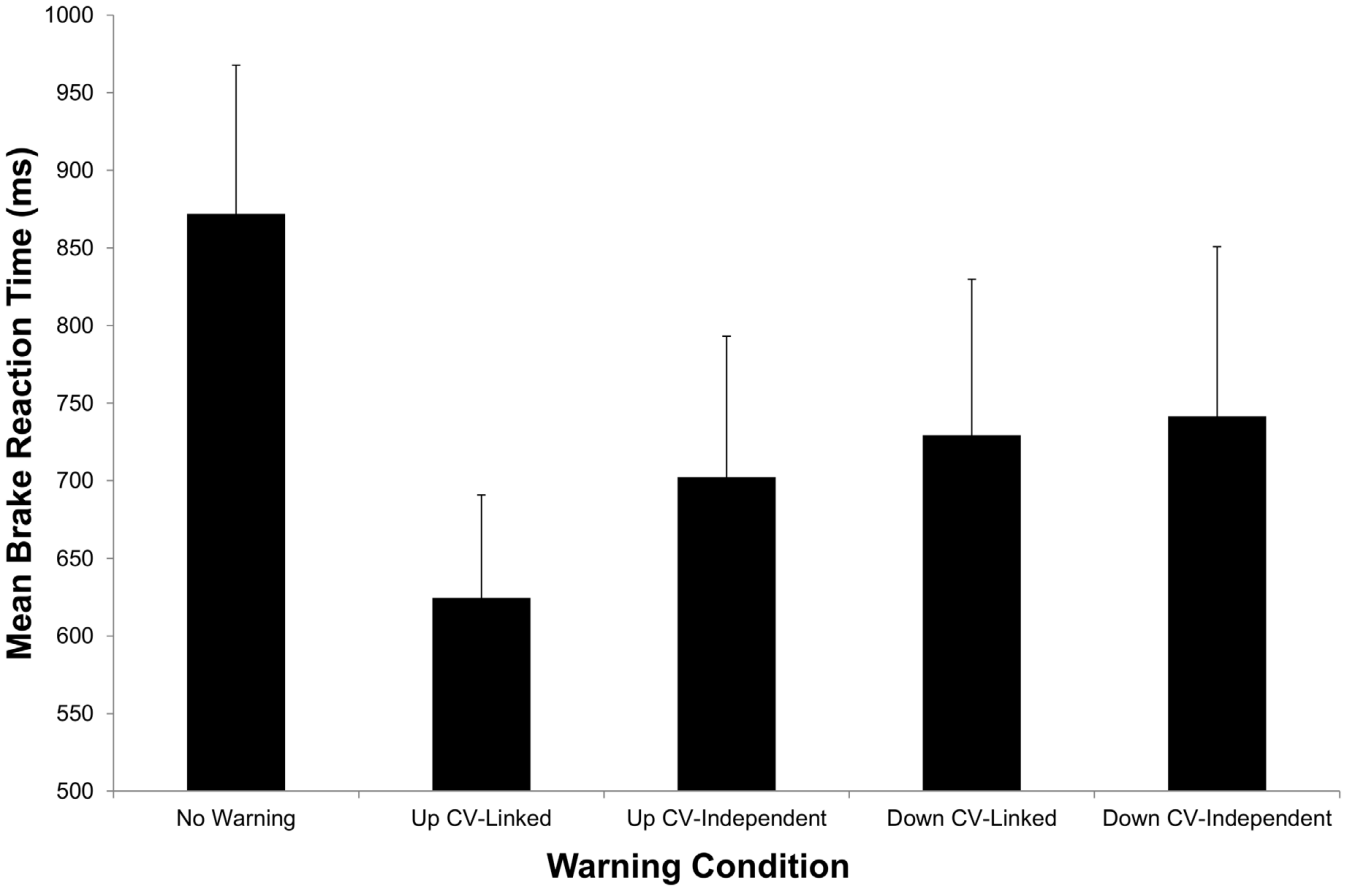
Mean braking reaction times for Experiment 2. Error bars are standard deviations.

Figure 2 shows the mean BRT for each of the four experimental conditions. The one-way ANOVA performed on these data revealed a significant effect of warning type: *F*(4, 56) = 14.8, *p*<0.001, 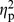 = .51. The planned comparisons revealed that BRTs were significantly faster for the up CV-linked warning as compared to all the other four warning conditions: no warning [*t*(14) = 7.3, *p*<0.001], up CV-independent [*t*(14) = 2.8, *p* = 0.01], down CV-linked [*t*(14) = 3.6, *p* = 0.003], and down CV-independent [*t*(14) = 3.8, *p* = 0.002]. Post-hoc pairwise comparison revealed that there was no significant difference between the two down warning types (p>0.5).

**Figure 3:**
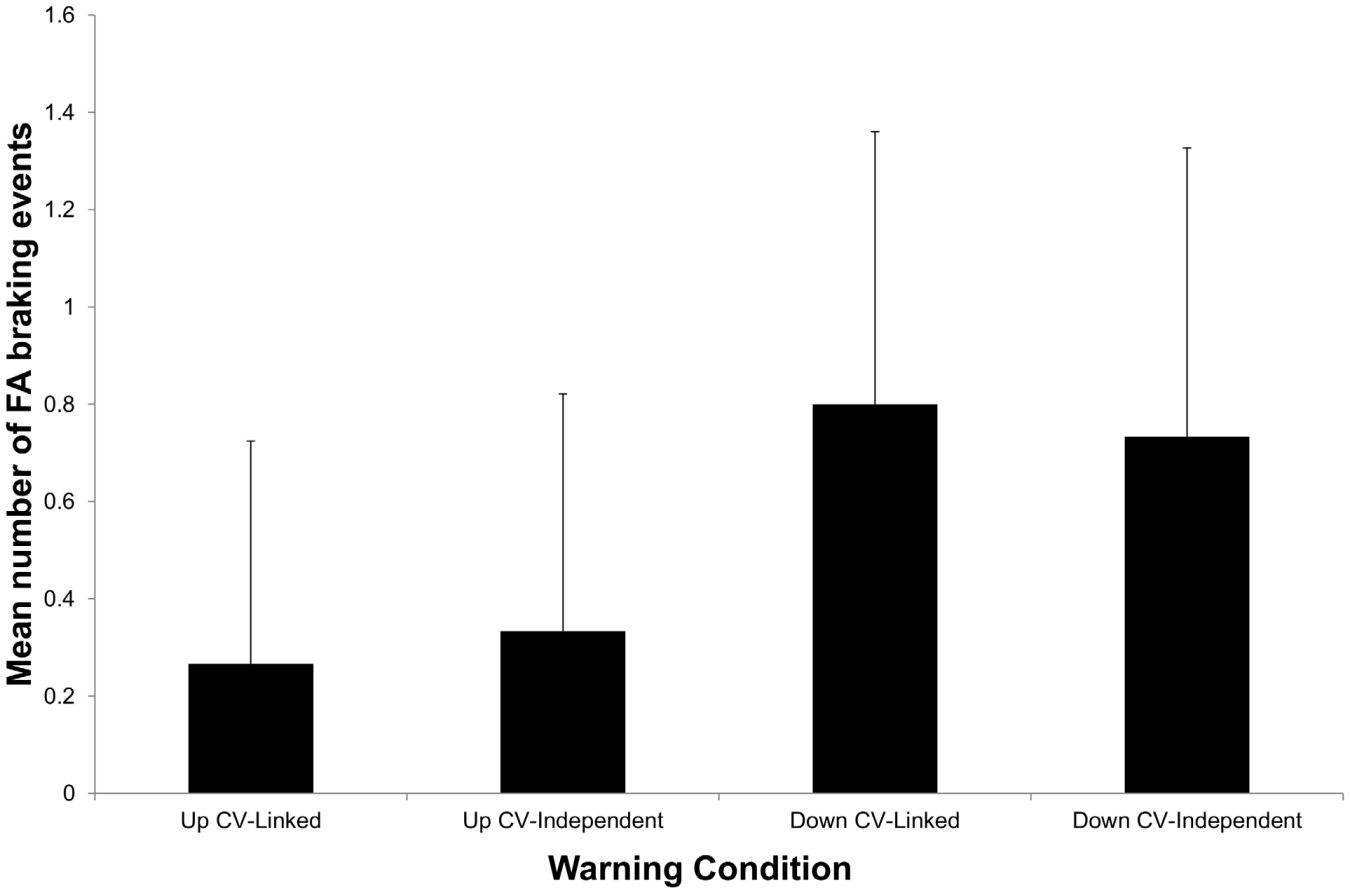
Mean number of false alarm braking events in Experiment 2. Error bars are standard deviations.

Figure 3 shows the mean total number of false alarm brake activations. The one-way ANOVA performed on these data revealed a significant effect of warning type: *F*(3, 42) = 4.1, *p* = 0.013, 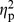 = .22. The planned comparisons revealed that the number of false alarm braking events was significantly lower for the up CV-linked warning as compared to both the down CV-independent [*t*(14) = 2.7, *p* = 0.013] and the down CV-linked [*t*(14) = 2.8, *p* = 0.014] warnings. There was no significant difference between the up CV-linked and up CV-independent warnings.

### Discussion

Our prediction had been that: (i) CV-linked warnings would lead to significantly faster BRTs as compared to CV-independent warnings (for both movement directions) and (ii) up CV-linked warnings would lead to significantly faster BRTs as compared to down CV-linked warnings. The results of Experiment 2 provide partial support for the first hypothesis: CV-linked warnings were significantly more effective when the motion direction was upwards but were not significantly different from CV-independent warnings when the motion direction was downwards. Therefore, consistent with the results of Experiment 1, it does not appear to be the case that linking a collision warning to closing velocity necessarily produces benefits over and above non-linked warnings – instead, it depends on other characteristics of the warning.

The BRT results from Experiment 2 were consistent with our second prediction: namely that the up CV-linked warning signal would lead to significantly faster BRTs as compared to the down CV-linked warning (and all other warning types). On the surface, this is consistent with the idea, described above, that warnings designed to convey some form of motion information will be more effective when they simulate motion towards an observer's head. But again note that it is not simply the directionality that produces the advantage as there was no difference between up CV-independent and down CV-independent warnings.

Interestingly, there was also a significant effect of warning type on false alarm brake activations in Experiment 2. In particular, as shown in Figure 3, there were a larger number of brake activations for the two down warnings (combined) versus for the two up warnings (combined): *t*(14) = −3.5, *p* = 0.003. We would argue that this effect occurred because the two warning directions had different effects on the perceptual-action system.

Previous research has shown that signals which indicate the direction of an object that a driver is about to collide with (e.g., a sound presented from the right side when a pedestrian is moving into the road from the right side) produce very different behavioural reactions as compared to those signals which indicate the direction in which the driver needs to go in order to avoid the object (e.g., a sound presented from the left side when a pedestrian is entering the road from the right side) [27], [28]. While the former signals appear to assist the driver in evaluating the collision event before acting, the latter signals appear to prime an immediate motor response. This effect can be seen in a study in which warning timing was varied [28]. When warnings were presented at relatively long TTC (i.e., when there was sufficient time for the driver to evaluate the event) signals presented in same direction as the object were more effective whereas for warnings presented at a relatively short TTC signals presented from the “escape direction” were more effective. We would argue that the present false alarm results are consistent with the idea that the downwards motion lead to response priming (i.e., it indicated the direction the driver needs to move their foot in order to avoid the collision) while the upwards motion did not.

## Discussion

Previous research has demonstrated that it is possible to create auditory forward collision warnings that are both abstract (i.e., they do not involve the use of naturally occurring stimuli) and informative (i.e., they convey information about the event that can be used to execute the appropriate action) ([7], [17] – Experiments 1 & 2). Our initial attempts to create an analogous warning using the sense of touch have produced somewhat unclear results as to the potential effectiveness of informative vibrotactile warnings ([17]-Experiment 3, [18]). The primary goal of the present study was to investigate whether the lack of effectiveness of the vibrotactile signals used in these previous studies was attributable to the fact that the parameters of the warning signals were not contingent on the parameters of the collision of event.

On the surface, the present study produced a mixed answer to this question. For the looming vibrotactile warning (Experiment 1) and the downwards apparent motion warning (Experiment 2), linking the rate of increase of warning intensity to CV did not result in the predicted benefit in terms of shortened BRTs. Conversely, for the upwards apparent motion warning (Experiment 2), the effect of CV-linking was as predicted with the up CV-linked warning having a substantially lower (by roughly 100 ms on average) BRT as compared to the other warnings tested. We next explore possible reasons for these seemingly discrepant results.

Before considering vibrotactile warnings in more detail, it is important to note that we have also found mixed results for different types of informative auditory collision warnings. In particular, we have found that CV-linked looming frequency warnings (i.e., an auditory signal that increased in frequency as the driver approached the lead vehicle; ([17] – Experiment 1) and CV-linked looming spatial warnings (i.e., an auditory signal that was presented from a larger area of space as the driver approached the lead vehicle; ([17] – Experiment 2) did not produce significantly lower BRTs as compared to non-informative auditory warnings. Furthermore, when either of these two signals was combined with the intensity looming warning [7] there was no additional BRT decrease over and above that produced by the intensity looming warning alone. These results therefore suggest that the benefits of informative collision warnings cannot be achieved by simply pairing any dynamic auditory signal with the collision event. Instead, there would appear to be signals that are more strongly associated with the approaching vehicle than others.

The results of the present study suggest that this is also the case for vibrotactile warnings. Both subjectively and based on BRT results, a looming intensity vibrotactile signal presented at a single body location does not appear to generate a percept of distal approaching motion. Conversely, apparent motion across the skin surface can more easily be associated with an object approaching the body. This effect only seems to occur when the direction of motion (towards the head) is compatible with the object motion direction, a result consistent with a previous touch research (as reviewed in [29]). It will be interesting in future research to more systematically investigate the stimulus parameters that are associated with effective distal attribution.

An alternative explanation for the effects observed in Experiment 2 of the present study is that the upwards and downwards motion signals had differential priming/cueing effects. It is possible that the up signals triggered an aversive/defensive response in the driver that prepared them for a faster avoidance response (i.e., braking) while the downwards cues may have drawn attention away from oneself leading to slower reaction time. It will be important for future research to investigate the relative contribution of distal attribution and response priming to these effects.

A final point to make concerning distal attribution is the potential for a learning effect. Previous research on tactile sensory substitution systems has shown that distal attribution for tactile stimulation only occurs after extensive practice (e.g., [30]). Therefore, it is possible that improved distal attribution (and possibly shortened BRT) may occur for all of the warnings used in the present study if drivers were given additional experience using them. However, given that in real driving collision warnings are meant to signal fairly rare events, the practical value of warning signal which requires training to be effective is questionable.

The findings of the present study were limited by the driving simulator paradigm used. First, the frequency of warning activations in the present study (once every 3 minutes) was much higher than would be expected to occur in real driving. It will be important for future research to determine whether the warnings used in the present study have the same effect when they occur infrequently. Second, because we used a fixed-based desktop simulator in the present study the whole body vibration associated with real driving was not present. Therefore, the signal-to-noise ratio of the vibrotactile warnings may have been unnaturally high (though see [6], for evidence to suggest that this does not influence the effectiveness of vibrotactile collision warnings). It will be important for future research to investigate the effectiveness of present warning in a motion based simulator and/or in real driving.
